# Efficacy and safety of montelukast sodium combined with fluticasone in the treatment of adult bronchial asthma: A protocol for systematic review and meta-analysis

**DOI:** 10.1097/MD.0000000000023453

**Published:** 2020-12-24

**Authors:** Huiling Luo, Hongmei Han, Xiaoli Liu, Qin Liu

**Affiliations:** a The Second People's Hospital of Lanzhou City, Lanzhou 730046, Gansu Province, China; b Fuling Central Hospital of ChongQing City, Chongqing 408000, China.

**Keywords:** bronchial asthma, fluticasone, montelukast sodium, protocol, randomized controlled trial

## Abstract

**Background:**

Bronchial asthma (BA) is a chronic airway inflammatory disease with reversible airflow limitation as the main clinical manifestations, such as wheezing, cough, shortness of breath, chest tightness, etc, mediated by a variety of inflammatory cells, which can be recurrent. Clinical can improve symptoms, but cannot be cured; glucocorticoid is the most important first-line medication. Clinical practice has shown that montelukast sodium combined with fluticasone in the treatment of adult BA can improve clinical efficacy and reduce adverse reactions. The purpose of this study is to systematically study the efficacy and safety of montelukast sodium combined with fluticasone in the treatment of adult BA.

**Methods:**

The Chinese databases (CNKI, VIP, Wanfang, Chinese Biomedical Database) and English databases (PubMed, the Cochrane Library, Embase, Web of Science) were searched by computer, for the randomized controlled clinical studies of montelukast sodium combined with fluticasone in the treatment of adult BA from establishment of database to October 2020. Two researchers independently extracted the relevant data and evaluated the quality of the literatures, and used RevMan5.3 software to conduct meta-analyze of the included literatures.

**Results:**

This study assessed the efficacy and safety of montelukast sodium combined with fluticasone in the treatment of adult BA through total effective rate, pulmonary function (FEV1, FVC, PEF, FEV1/FVC), and adverse reactions.

**Conclusion:**

This study will provide reliable evidence-based evidence for the clinical application of montelukast sodium combined with fluticasone in the treatment of adult BA.

**OSF Registration number:**

DOI 10.17605/OSF.IO/CKQFM

## 1 Introduction

Bronchial asthma (BA) is a chronic inflammatory disease of the airway mediated by many inflammatory cells, such as eosinophils, mast cells, T lymphocytes,^[1,2]^ which often leads to airway hyperresponsiveness, extensive and variable reversible airflow limitation, and causes recurrent symptoms such as wheezing, expiratory dyspnea, chest tightness, or cough. It affects 10% of the world's population and creates a serious social and economic burden. Children and adolescents are more likely to suffer from,^[3]^ adults are also vulnerable, and it repeatedly attacks, affecting work and life, causing great distress to adults. The main drugs used in BA are glucocorticoid,β2 receptor agonist, anticholinergic agent, theophylline, leukotriene receptor antagonist, mast cell membrane stabilizer and others.^[4]^ Because the symptoms of acute attack of BA are serious, the medicine is required to have the characteristics of quick onset and good curative effect. Inhaled glucocorticoid can enhance local anti-inflammatory effect of airway and reduce systemic effect, so it is the most effective drug for asthma. However, BA attacks repeatedly and is difficult to cure, and long-term use of hormones will lead to certain adverse reactions,^[5]^ therefore, the current clinical treatment of BA is mostly glucocorticoid combined with other drugs. Among them, montelukast sodium is an oral selective leukotriene receptor antagonist, which plays an anti-inflammatory role by regulating the biological activity of leukotriene and relaxes bronchial smooth muscle.^[6]^ In the clinical treatment of BA, the use of montelukast sodium combined with inhaled glucocorticoid can better fuse the mechanism of the 2 drugs and better inhibit the production of inflammation. Finally achieve the anti-inflammatory effect, to achieve the relief of symptoms of patients. Studies shows that montelukast sodium combined with fluticasone has a better clinical effect in adult BA.

At present, there are many randomized controlled studies,^[7–10]^ which show that montelukast sodium combined with fluticasone in the treatment of adult BA can effectively improve the clinical efficacy, control the symptoms of clinical acute attack, improve the lung function of patients, and have high clinical application value. However, there are differences in the research scheme and curative effect of each clinical trial, which leads to the uneven results, which to some extent affects the promotion of the therapy. Therefore, this study plan systematically evaluated the efficacy and safety of montelukast sodium combined with fluticasone in the treatment of adult BA. It provides a reliable reference for the clinical application of montelukast sodium combined with fluticasone in the treatment of adult BA.

## 2 Methods

### 2.1 Protocol register

This protocol of systematic review and meta-analysis has been drafted under the guidance of the preferred reporting items for systematic reviews and meta-analyses protocols (PRISMA-P). In addition, it has been registered on open science framework (OSF) on October 25, 2020. (Registration number: DOI 10.17605/OSF.IO/CKQFM).

### 2.2 Ethics

As the protocol does not require patient recruitment and personal information collection, it does not require approval from an ethics committee.

### 2.3 Eligibility criteria

#### 2.3.1 Types of studies

We will collect all available randomized controlled trials (RCTs) on montelukast sodium combined with fluticasone in the treatment of adult BA, regardless of blinding, publication status, and region; however, the language will be limited to only Chinese and English.

#### 2.3.2 Research subjects

Adult patients with definite diagnosis of BA,^[11]^ excluding pregnant or lactating women, patients complicated with malignant tumor, patients complicated with severe heart, brain, kidney, lung, liver diseases, and other diseases. Nationality, race, gender, and course of illness are not limited.

#### 2.3.3 Interventions

The treatment group and the control group were given routine basic treatment, including bed rest, cough and phlegm elimination, oxygen therapy, and appropriate anti-infection treatment for patients with complicated infection. The control group was treated with fluticasone at the same time, and the treatment group was treated with montelukast sodium combined with fluticasone.

#### 2.3.4 Outcome indicators

(1) Primary outcome: ① the overall effective rate. Total effective rate = (cure number + effective number)/ total number ∗100%. Cure: BA symptoms basically disappear, occasionally mild disease can be alleviated without medication, peak expiratory flow (PEF) diurnal fluctuation <20%, PEF or forced expiratory volume in the first second (FEV1) not less than 80% after treatment. Significant: symptoms are significantly reduced compared with that before treatment, PEF diurnal fluctuation <20%, PEF or FEV1 reached 60% to 79% of the predicted value after treatment, and drug treatment is still needed when the disease occurred. Improve: BA symptoms are alleviated to a certain extent. After treatment, PEF or FEV1 reach the predicted value of 45% to 55%, requiring bronchodilators or glucocorticoids. Invalid: no improvement in clinical symptoms and no change in PEF or FEV1 detection values, or even deterioration of the condition.
(2) Secondary outcomes: ① pulmonary function [FEV1); forced vital capacity (FVC); (PEF); ratio of forced expiratory volume to forced vital capacity in the first second (FEV1/FVC)]; ② recurrence rate; ③ incidence of adverse reactions; ④ quality of life of patients.

### 2.4 Exclusion criteria

(1) Duplicate published papers;
(2) Articles published as abstracts or with incomplete data and unable to obtain complete data after contacting the author;
(3) Studies in which data are clearly wrong;
(4) Studies in which interventions combined with other therapies, such as traditional Chinese medicine, acupuncture and moxibustion;
(5) Studies in which interventions with other glucocorticoids;
(6) Studies with no related outcome indicators.

### 2.5 Search strategy

“meng lu si te na”(Montelukast sodium), “Fu ti ka song”(Fluticasone), “zhi qi guan xiao chuan”(bronchial asthma), and other Chinese search words were searched in Chinese databases, including CNKI, VIP, Wanfang Data Knowledge Service platform, and China biomedical Database. English retrieved words such as “Montelukast sodium,” “Fluticasone,” “bronchial asthma,” etc, were searched in English databases, including PubMed, the Cochrane Library, EMBASE, and Web of Science. The retrieval time was from the establishment of the database to October 2020, and all domestic and foreign literatures on montelukast sodium combined with fluticasone for the treatment of adult BA were collected. Take PubMed as an example, and the retrieval strategy is summarized in Table 1.

**Table 1:** PubMed database retrieval strategy.

Number	Search terms
#1	Montelukast Sodium [Mesh]
#2	Singulair[Mesh]
#3	Montelukast [Title/Abstract]
#4	Montelukast Sodium Tablets [Title/Abstract]
#5	#1 OR #2 OR #3 OR #4
#6	Bronchial asthma [Mesh]
#7	Asthma [Mesh]
#8	Bronchus asthma [Title/Abstract]
#9	Bronchil sthm [Title/Abstract]
#10	#6 OR #7 OR #8 OR #9
#11	#5 AND #10

### 2.6 Data screening and extraction

**Figure 1:**
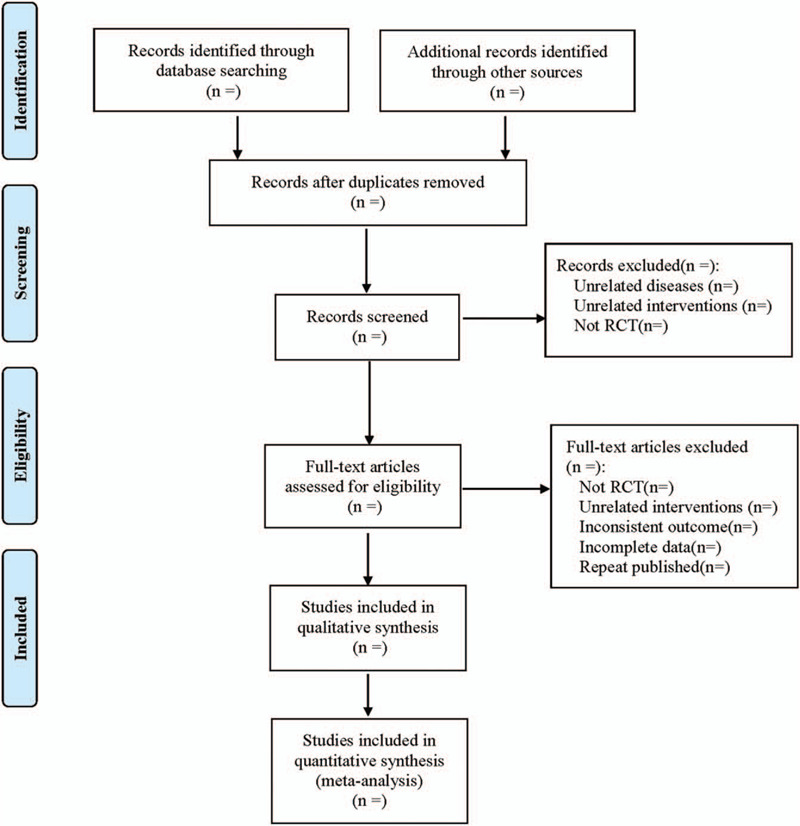
The process of literature filtering.

Referring to the research selection method in the 5.0 edition of the Cochrane collaborative network system evaluator manual, according to the PRISMA flow chart, 2 researchers read the topics and abstracts independently. First exclude RCT, which clearly does not meet the inclusion criteria. Read the full text carefully for studies that may meet the inclusion criteria and determine the final RCT. Two researchers cross-check each other to include the results. In case of disagreement in the screening process, the 2 parties shall discuss and resolve, if not, listen to the opinions of third parties and reach an agreement. Excel2013 was also used to extract relevant information, including: ① Clinical studies (title, first author, year of publication, sample size, sex ratio, mean age, mean course, stage); ② Interventions (Type, dosage, frequency, course of treatment of conventional symptomatic support therapy in treatment group and control group; Dosage, frequency, course of treatment of fluticasone in treatment group and control group; Dose, frequency, course of treatment of montelukast sodium); ③ Risk bias assessment elements in randomized controlled trials; ④ Outcome indicators. The literature screening process is shown in Figure 1.

### 2.7 Literature quality assessment

By using Cochrane Handbook 5.1.0 evaluation criteria, the quality of the literature was evaluated from 5 aspects: inclusion bias, scheme bias, measurement bias, follow-up bias, and score bias. If these 5 indexes are consistent, the quality of the literature is high, otherwise the bias may be generated. On the basis of the performance of the literature included in the above evaluation items, the 2 researchers will give low-risk, unclear, or high-risk judgments and cross-check each after completion. If there are differences, discussions will be held. If there is no agreement between the 2, it will be discussed with third-party researchers.

### 2.8 Statistical analysis

#### 2.8.1 Data analysis and processing

Use RevMan5.3 software provided by Cochrane collaboration for statistical analysis. ① Relative risk (RR) is taken as the statistic of the dichotomous variable. For continuous variables, when the tools and units for measuring indicators are the same, choose the weighted mean difference (WMD), but when the tools and units for measuring indicators are different, choose standardized mean difference (SMD). All the above are represented by effect value and 95% confidence interval (95% CI), respectively. ② Heterogeneity test: the *I*^*2*^ value is used to quantitatively evaluate the heterogeneity between studies. The variation between different studies in systematic evaluation is called heterogeneity. If *I*^*2*^ ≤50%, that the heterogeneity is good, the fixed effect model is adopted. If *I*^*2*^ >50%, it is considered to be significant heterogeneity, the source of heterogeneity will be explored by subgroup analysis or sensitivity analysis. If we cannot analyze the source of heterogeneity, random effect model can be used to analyze. If there is significant clinical heterogeneity between the 2 groups, and grouping analysis is not available, descriptive analysis will be used.

#### 2.8.2 Dealing with missing data

If there are missing data in the article, contact the author via email for additional information. If the author cannot be contacted, or the author has lost relevant data, descriptive analysis will be conducted instead of meta-analysis.

#### 2.8.3 Subgroup analysis

Subgroup analysis was carried out according to age group: youth, middle age and old age, and subgroup analysis was carried out according to course of treatment.

#### 2.8.4 Sensitivity analysis

In order to test the stability of meta-analysis results of indicators, a one-by-one elimination method will be adopted for sensitivity analysis.

#### 2.8.5 Assessment of reporting biases

Funnel plots were used to assess publication bias if no fewer than 10 studies were included in an outcome measure. Moreover, Egger and Begg tests were used for the evaluation of potential publication bias.

#### 2.8.6 Evidence quality evaluation

The Grading of Recommendations Assessment, Development, and Evaluation (GRADE) will be used to assess the quality of evidence. It contains 5 domains (bias risk, consistency, directness, precision, and publication bias). And the quality of evidence will be rated as high, moderate, low, and very low.

## 3 Discussion

BA is the result of reduced immune system due to exposure to allergens or environmental exposure.^[12]^ Typical asthma attack symptoms are easy to identify, but the etiology of asthma is complex, its attack and the body's reactivity, that is, genetic factors and atopic quality of individual differences, allergens, and stimuli of different quality and quantity, can lead to asthma attack symptoms of ever-changing. Sick people, especially adults, may gradually lose their ability to work, which places a huge economic burden on the health care system and society. According to the latest China Pulmonary Health Research (CPH) survey in 2019, the prevalence of asthma in people over 20 years (including 20 years old) in China is 4.2%, and the total number of patients is 45.7 million, which is far more than expected.^[13]^ Studies have shown that adult-onset asthma (AOA) has a worse prognosis than childhood-onset asthma (COA).^[14]^ Therefore, the treatment of adult BA has received more and more attention. According to previous studies, the occurrence of the disease is not only related to a variety of cell-mediated inflammatory reactions, but also involved in neuroregulation and immune response. Eosinophils, mast cells, T lymphocytes, neutrophils, smooth muscle cells, airway epithelial cells, leukotriene, interleukin, and other components are involved in mediating the inflammatory response.^[15–17]^ Glucocorticoids are the most effective first-line drugs for asthma. Its mechanism is to inhibit the metabolism of arachidonic acid, reduce the synthesis of leukotriene and prostaglandin, activate and improve the responsiveness of airway smooth muscle to β2 receptors, inhibit the chemotaxis and activation of eosinophils, and inhibit cytokine synthesis, reduce microvascular leakage. However, long-term or heavy use or intravenous drip can cause electrolyte disorders, interfere with the body's immunity and other side effects, such as hoarseness, candida oropharynx infection, osteoporosis, increased cortical acid and other adverse reactions. Inhalation can make the drug contact the airway in the largest area and improve the affinity of glucocorticoid receptor. At present, the commonly used clinical drugs are budesonide, momethasone, fluticasone-formoterol inhalants, and so on.^[18–20]^ Leukotriene antagonists are Cys-LT competitive antagonists that reduce inflammation and Th2 response by blocking Cys-LT. Leukotriene receptor antagonists (LTRA) have been recommended for the treatment of persistent asthma, representing drugs such as montelukast sodium.^[21]^ Clinical use of inhaled glucocorticoid fluticasone combined with montelukast sodium in the treatment of adults BA can improve lung function, improve the quality of life of patients, and reduce adverse reactions caused by long-term use of hormones, has achieved good clinical effect.

The clinical efficacy of montelukast sodium combined with fluticasone in the treatment BA adults is reliable. However, the evidence from RCTs is inconsistent. With the increasing number of clinical trials, it is urgent to systematically evaluate montelukast sodium combined with fluticasone in the treatment of adult BA. In this study, we will summarize the latest evidence of the efficacy of montelukast sodium combined with fluticasone in adult BA. This work also provides useful evidence for determining the efficacy and safety of montelukast sodium combined with fluticasone in the treatment of adult BA patients, which is beneficial for both clinical practice and health-related decision makers. However, this systematic review has some limitations. There may be some clinical heterogeneity in the way of routine western medicine therapy and the degree of patient's condition. The course of disease is also different, and may have a certain impact on the results. Due to the limitation of language ability, we only search English and Chinese literature, and may ignore the research or report of other languages.

## Author contributions

**Data collection:** Huiling luo and Hongmei Han.

**Funding support:** Qin Liu.

**Literature retrieval:** Huiling luo and Hongmei Han.

**Software operating:** Xiaoli Liu.

**Supervision:** Qin Liu.

**Writing – original draft:** Huiling luo and Hongmei Han.

**Writing – review and editing:** Huiling luo and Qin Liu.

**Data curation:** Huiling luo, Hongmei Han.

**Funding acquisition:** Qin Liu.

**Software:** Xiaoli Liu.

**Writing – original draft:** Huiling luo, Hongmei Han.

**Writing – review & editing:** Qin Liu.
